# Hypercupremia in female munitions workers using taking oral contraceptives: a case series

**DOI:** 10.47626/1679-4435-2021-638

**Published:** 2021-04-30

**Authors:** Maurício Petroli, Assad Charbel Chequer Bon-Habib, Aline de Souza Espindola Santos, Carmen Ildes Rodrigues Fróes Asmus, Angélica dos Santos Vianna

**Affiliations:** 1 Faculdade de Medicina, Universidade Federal do Rio de Janeiro (UFRJ), Rio de Janeiro, RJ, Brazil; 2 Ambulatório de Toxicologia Clínica Ambiental e Ocupacional, Hospital Clementino Fraga Filho and Instituto de Estudos em Saúde Coletiva, UFRJ, Rio de Janeiro, RJ, Brazil

**Keywords:** copper, contraceptives, occupational exposure

## Abstract

Copper is an essential trace element for homeostasis and is mostly obtained through the diet. Copper can also enter the body through occupational and accidental exposure, resulting in the elevation of serum copper levels (hypercupremia). Other factors associated with hypercupremia include smoking, use of oral contraceptives, and several clinical conditions. This case series describes the presence of hypercupremia in workers exposed to copper while also taking oral contraceptives. Serum copper levels of the sample remained high, even after participants spent time away from work, normalizing only after a change in contraceptive methods. The present results underscore the importance of considering oral contraceptives as a possible cause of hypercupremia in women with occupational exposure to copper, regardless of symptomatic status.

## INTRODUCTION

Copper is a ubiquitous element in the environment. It is an essential trace element and the third most abundant in the body after zinc and iron. It binds mainly to ceruloplasmin, and to a lesser extent, albumin so that it circulates freely in the blood. Copper has several essential functions in the body. It helps combat oxidative stress (as a constituent of antioxidant enzymes) and is involved in the synthesis of hemoglobin (iron mobilization), connective tissue (formation of collagen and elastin), adrenaline, and thyroid hormones, in addition to being the cofactor of metalloenzymes involved in carbohydrate and lipid metabolism.^1,2^

The mean daily intake of copper in adults is approximately 1 mg (ranging from 0.9 to 2.2 mg), mostly from dietary sources.^1,3^ The total amount of copper in the body is estimated to range from 50 to 120 mg, with the highest concentrations found in bile, the liver, the brain, the heart, and the kidneys.^1^ Elevated copper levels, or hypercupremia, can occur in clinical conditions such as Menkes disease, thyrotoxicosis, and Wilson’s disease.^4^ Healthy women show an increase in serum copper levels during pregnancy, and this variation is considered normal.^5^ Lifestyle habits such as tobacco and alcohol consumption are also associated with alterations in copper levels.^6^

Workers in the mining and smelting industries, and those engaged in activities such as welding, painting, and farming, have also been known to develop acute or chronic copper toxicity.^1^ Copper intoxication can be intentional, as in the case of copper sulfate poisoning, or accidental, caused by consuming food that has been cooked or stored in copper pots, water piped through copper plumbing, and the use of intrauterine devices (IUDs) or oral contraceptives (OC).^7^

When investigating the cause of hypercupremia, professionals should consider the possibility of accidental ingestion, as well as occupational or environmental exposure to copper. Patients should also be screened for Wilson’s disease. However, if the patient uses OC, this should also be closely examined since these medications are associated with hypercupremia. The present study reported on the cases of 4 patients with environmental and occupational exposure to copper, who nevertheless presented with hypercupremia as a result of OC use.

## CASE REPORTS

The study involved 4 female patients, born and residing in Rio de Janeiro. The women were of reproductive age (47, 38, 34, and 21 years, respectively, hereafter referred to as P1, P2, P3, and P4), previously healthy, and worked in a naval munitions factory (median length of current employment; 5.5 years). The women worked in quality control, inspecting the products and conducting tests that included explosions to test the integrity of the devices. Participants worked for 8 hours a day, 5 days a week. The main environmental risks to which they were exposed at work included physical factors such as noise and chemical contaminants such as lead, copper, brass, black powder, tetrachloroethylene, and toluene. All workers used personal protective equipment (gloves, masks, and hearing protection) and underwent six-monthly health checks, which included physical exams, audiometry, complete blood counts, liver function tests, measurements of lead and copper levels in the blood, and urine tests for methylhippuric and trichloroacetic acid, both of which are metabolites of tetrachloroethylene and toluene.

In one of these assessments, all 4 women displayed elevated copper serum levels, although all remaining exams were normal. The women were initially placed on a 15-day leave. However, their copper levels did not change, and they were then referred for specialized assessment at the Clinical Environmental and Occupational Toxicology Service of the Hospital Universitário Clementino Fraga Filho at the Universidade Federal do Rio de Janeiro.

Upon admission, three patients were asymptomatic (P1, P2, and P3), and one (P4) reported symptoms such as headaches, fatigue, and generalized pain, especially in the ankle joints. Clinical history interviews revealed no commonalities between patients’ places of residence, medical history, or family history of illness. None of the patients were pregnant or reported any previous illness. One (P1) was a smoker, and all denied alcohol abuse. All participants used estrogen-based OC and denied using other medications.

Patients were in adequate overall health, and the physical examinations of three patients (P1, P2 and P3) revealed normal findings. The symptomatic patient (P4), however, was pale (1/4+) and had ankle arthritis. After 15 days away from work, participants were retested by the same laboratory, and continued to display elevated copper levels in the blood (colorimetry method) (P1: 324 mcg/dL; P2: 275.1 mcg/dL; P3: 240.1 mcg/dL; P4: 271 mcg/dL; reference value: 80-155 mcg/dL). This was considered the baseline assessment for the present study (T1). All other laboratory tests (basic biochemical profile and complete blood count) were normal.

Participants were therefore advised to stay home from work for an additional month, after which laboratory tests were performed to evaluate liver and kidney function, both of which tend to be affected by copper toxicity. The results of these tests were normal. P4, specifically, underwent additional testing to screen for collagen diseases and hyperuricemia. Both tests were negative.

At this point, the company where participants worked was also asked to provide an occupational safety report, specifying the cooper levels in the occupational environment. However, no such report was provided.

Tests performed after a 3-month period (T2) revealed that serum copper levels in the sample remained high, even though participants were still away from work (P1: 232 mcg/dL; P2: 232.4 mcg/dL; P3: 252.2 mcg/dL; P4: 271 mcg/dL). Once clinical evaluation excluded other causes of hypercupremia, it was hypothesized that OC, which all patients used at the time of the study, was responsible for the elevated copper levels.

Each patient’s gynecologist was then asked to change their contraceptive method.

Three patients (P1, P2, and P3) followed this recommendation, and after 2 months (T3), showed a marked decrease in serum copper levels, which then fell in the normal range (P1: 142 mcg/dL; P2: 122 mcg/dL; P3: 140.2 mcg/dL). Once these results were obtained, participants were medically cleared to return to work. Two participants were transferred to a sector with no risk of exposure, while one returned to her former role. Participants continued to be monitored on an outpatient basis, and their serum copper levels remained normal according to laboratory examinations performed after 6 months (T4) (P1: 125 mcg/dL; P2: 135 mcg/dL; P2: 115.6 mcg/dL).

One of the patients (P4), however, opted not to change her OC and continued to have joint pain and hypercupremia, although her serum copper levels had decreased by 16% (271 mcg/dL [T1] to 227 mcg/dL [T3]). The patient was referred for assessment at a rheumatology clinic and, although she was advised not to return to work, she did so regardless. The patient (P4) did not continue clinical follow-up.

**Figure 1:**
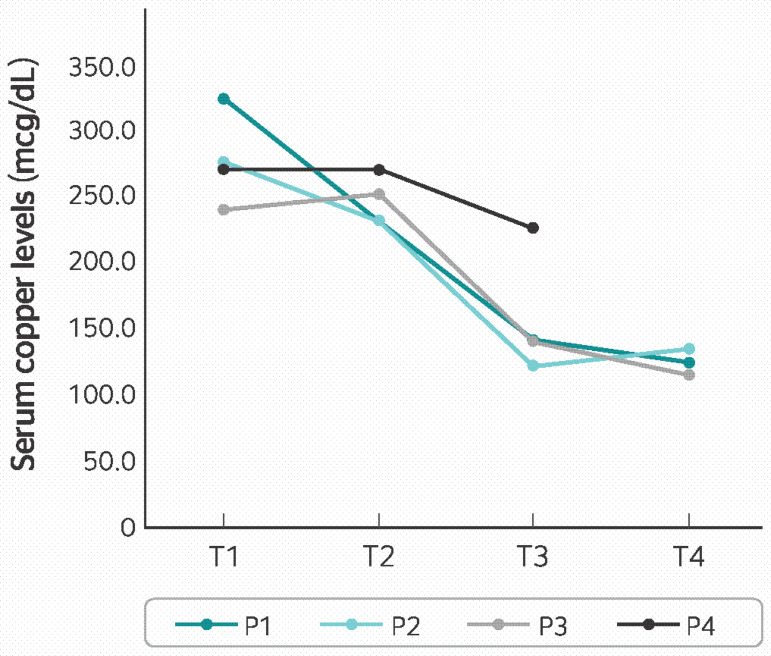
Total serum copper levels of 4 patients (referred to as P1, P2, P3 and P4) during clinical follow-up; T1 on admission, T2 after 3 months, T3 after 2 months of discontinuing oral contraceptive (except for P4) and T4 after 6 months. Reference values for copper in women: 80 to 50 mcg/dL.

The changes in copper serum levels for all 4 patients (T1, T2, T3, and T4) over time are shown in Figure 1.

## DISCUSSION

All patients in the present study had been exposed to copper at their workplace and through the use of estrogen-based OC (environmental exposure). The exposure in occupational settings was due to the handling of munitions, which contain lead, copper/tin alloys, and brass (copper and zinc) alloys. As a result of manual labor and the testing of these devices, workers came into contact with the metal through their respiratory and digestive tracts.^8,9^

Occupational exposure to copper can have toxic effects on the body, and copper serum levels are considered potential biomarkers of effect rather than markers of exposure due to individual differences in toxicokinetics.^10^ On the other hand, OC have been associated with increased serum copper levels, which normalize after the medication is interrupted.^11,12^

The main diagnostic hypothesis for patients in the present study was hypercupremia due to occupational exposure. It is important to note that, while patients were evaluated every six months, at which point their serum copper levels were also examined, Brazilian legislation (Norm NR-07) does not establish the frequency of biological monitoring, the examinations to be performed, or the reference values for the interpretation of these exams. The scientific literature recommends the analysis of 24 h blood and urine samples, providing reference values of 80 to 150 mcg/dL and 15 to 60 mcg/24 h for each test, respectively.^13^

At first, the persistence of high cooper levels was attributed to the fact that patients may not have been away from work long enough to affect the half-life of copper ranges from 13 to 33 days.^14^ However, this hypothesis was not borne out since the levels remained elevated even after a 3-month leave, with the lowest result in the sample exceeding the reference value by 49%. The alternative hypothesis adopted was another cause of hypercupremia which has been described in the literature: the use of OC.^15^ After changing contraceptive methods, three patients experienced normalization of copper levels. This was not observed, however, in the patient who did not follow this medical recommendation. This finding corroborated the hypothesis of OC as the cause of hypercupremia. In the scientific literature, only two case reports have described similar situations. The first was conducted in Spain and described two young patients with hypercupremia whose copper serum levels returned to normal after they discontinued OC.^11^ The second report described two patients in the United States who had pigmented corneal rings and hypercupremia associated with the use of OC. The discontinuation of OC led to the normalization of copper serum levels and reduction of pigmented rings in both patients.^12^

Although the literature has not specified the time required for the normalization of hypercupremia after discontinuation of OC, some authors suggest that this should occur in 3 to 4 weeks. However, in the second of the previously cited studies, this only occurred after 60 days.^12^ It is important to note that, although the patient who did not change contraceptive methods continued to show elevated copper levels, which far exceeded the reference values, these levels decreased by 16% throughout the course of the study, likely due to the time spent away from work.

The hypercupremia associated with OC seems related to the elevation of serum ceruloplasmin, the primary transporter of copper in the body, which occurs due to estrogen regardless of dose.^7^ OC alter the plasma concentration of zinc and copper, promoting oxidative stress and inducing the elevation of ceruloplasmin levels.^7^ In the present study, serum ceruloplasmin and zinc levels were not evaluated. The progestogens in OC probably modulate copper metabolism, as evidenced by the higher copper levels observed in women using OC containing antiandrogenic progestogens.^15^ These drugs increase copper levels by an average of 57 mcg/dL (95% confidence interval: 49-66 mcg/dL), though these values tend to remain below 200 mcg/dL.^7^

The clinical outcomes associated with elevated copper levels vary widely, ranging from an asymptomatic or oligosymptomatic course with nonspecific symptoms to liver damage (jaundice), kidney damage (hematuria, anuria), and neurological issues (coma).^7^ According to the literature, serum concentrations greater than 300 mcg/dL are associated with gastrointestinal symptoms, while values over 500 mcg/dL lead to severe intoxication or death.^9^ In this study, three patients remained asymptomatic, and one showed nonspecific symptoms and joint pain, though subsequent investigation excluded the presence of rheumatological disease, specifically rheumatoid arthritis. It is important to note that copper has been investigated as a risk factor for this condition.^16^ On the other hand, studies have also shown that elevated copper levels may be a marker of clinical activity for this illness.^17^

This case series indicates that elevated serum copper levels require a complex differential diagnosis and demand extensive knowledge of professionals’ possible causes. The clinical investigation should consider the patient’s clinical and occupational history, and the reported use of OC in women of reproductive age. Lastly, although the association between OC and hypercupremia has been demonstrated in the literature, few epidemiological studies have examined this issue. Since OC are among the main contraceptive methods used by women, awareness of their relationship to copper levels may allow health care professionals to conduct more thorough assessments in clinical and occupational settings, especially when treating women exposed to copper, in addition to potentially simplifying the clinical investigation process.

This is the first case series to address hypercupremia in women with OC use and occupational exposure to copper in Brazil. Various factors can cause elevated cooper serum levels, and the differential diagnosis may require complementary examinations, some of which can be costly - as such, acknowledging OC as a possible cause can simplify the investigation. We, therefore, recommend that health professionals consider the role of OC when performing the biological monitoring of women with occupational exposure to copper.
